# Dietary Antioxidant Micronutrients and All-Cause Mortality: The Japan Collaborative Cohort Study for Evaluation of Cancer Risk

**DOI:** 10.2188/jea.JE20170023

**Published:** 2018-09-05

**Authors:** Enbo Ma, Hiroyasu Iso, Kazumasa Yamagishi, Masahiko Ando, Kenji Wakai, Akiko Tamakoshi

**Affiliations:** 1 Department of Clinical Trial and Clinical Epidemiology, University of Tsukuba Faculty of Medicine, Ibaraki, Japan; 2 Public Health, Department of Social Medicine, Osaka University Graduate School of Medicine, Osaka, Japan; 3 Department of Public Health Medicine, University of Tsukuba Faculty of Medicine, Ibaraki, Japan; 4 Center for Advanced Medicine and Clinical Research, Nagoya University Graduate School of Medicine, Aichi, Japan; 5 Department of Preventive Medicine, Nagoya University Graduate School of Medicine, Aichi, Japan; 6 Department of Public Health, Hokkaido University Graduate School of Medicine, Hokkaido, Japan

**Keywords:** dietary antioxidants, carotenoids, vitamin C, vitamin E, all-cause mortality

## Abstract

**Background:**

Oxidative stress, the imbalance between pro- and antioxidants, has been implicated in the etiology and pathophysiology of the incidence and mortality of many diseases. We aim to investigate the relations of dietary intakes of vitamin C and E and main carotenoids with all-cause mortality in Japanese men and women.

**Methods:**

The Japan Collaborative Cohort Study for Evaluation of Cancer Risk had 22,795 men and 35,539 women, aged 40–79 years at baseline (1988–1990), who completed a valid food frequency questionnaire and were followed up to the end of 2009.

**Results:**

There were 6,179 deaths in men and 5,355 deaths in women during the median follow-up of 18.9 years for men and 19.4 years for women. Multivariate hazard ratios for the highest versus lowest quintile intakes in women were 0.83 (95% confidence interval [CI], 0.76–0.90; *P* for trend < 0.0001) for vitamin C, 0.85 (95% CI, 0.78–0.93; *P* for trend < 0.0001) for vitamin E, 0.88 (95% CI, 0.81–0.96; *P* for trend = 0.0006) for β-carotene, and 0.90 (95% CI, 0.82–0.98; *P* for trend = 0.0002) for β-cryptoxanthin. The joint effect of any two of these highly correlated micronutrients showed significant 12–17% reductions in risk in the high-intake group compared with the low-intake group in women. These significant associations were also observed in the highest quintile intakes of vitamin C, vitamin E, and β-carotene in female non-smokers but were not observed in female smokers, male smokers, and non-smokers.

**Conclusions:**

Higher dietary intakes of antioxidant vitamins may reduce the risk of all-cause mortality in middle-aged Japanese women, especially female non-smokers.

## INTRODUCTION

Oxidative stress, the imbalance between pro- and antioxidants, has been implicated in the etiology, pathophysiology, and increased incidence of many chronic diseases and mortality.^1^^,^^2^ Vitamin C, vitamin E, and carotenoids are essential antioxidants in diet and may prevent oxidative damages by free radicals.^3^^–^^5^ Observational studies in populations reported that higher dietary intake of vitamin C,^6^^–^^9^ vitamin E,^10^ and carotenoids,^7^^,^^8^^,^^10^ or greater balance of antioxidant scores^4^^,^^11^ have been associated with decreased risk of all-cause mortality; however, these relationships were not confirmed in many studies for individual dietary nutrients, such as vitamin C,^2^^,^^5^^,^^10^^,^^12^^,^^13^ vitamin E,^5^^,^^7^^,^^12^^,^^13^ and β-carotene.^5^^,^^12^^,^^13^ In addition, smokers can be at risk for anti-/pro-oxidant imbalances of body tissues due to the excessive oxidants and free radicals from cigarette smoke.^11^

Fewer studies have been carried out to measure the association between antioxidants and the risk of all-cause mortality in Asian populations.^9^ Antioxidants and other micronutrients are rich in Japanese foods, such as fruits, vegetables, and green tea.^14^ The JACC Study reported that higher dietary intake of potatoes, spinach, or garland chrysanthemums was associated with reduced risk of all-cause mortality in both men and women, and that higher intake of carrot or pumpkin was associated with reduced risk of all-cause mortality in women.^15^ Favorable effects of fruits and vegetables could in part be driven by high antioxidant nutrients.^16^ The JACC Study also reported inverse associations of dietary vitamin C and E intakes with mortality from cardiovascular disease (CVD).^14^ Although that report did not include carotenoids, serum beta-carotene was associated with cancer and all-cause mortality in a subpopulation of the JACC Study.^17^^,^^18^ In spite of these reports, the overall association between dietary nutrients and all-cause mortality has not been comprehensively examined in the JACC Study. In this report, we aim to assess the relations of individual dietary intakes of antioxidant nutrients, namely vitamin C, vitamin E, and main carotenoids, as well as additive or synergistic properties in the combination of them, with all-cause mortality in Japanese men and women. Furthermore, we examined effect modification by smoking in groups defined according to dietary micronutrient intakes.

## METHODS

### Study cohort

The JACC Study is a cohort study that comprised a nationwide community-based sample of 110,585 persons (46,395 men and 64,190 women), aged 40 to 79 years during the baseline period (1988–1990), from 45 communities of Japan. Participants completed self-administered questionnaires about their lifestyles and medical histories of previous CVD or cancer. Previous publications have described the methods in detail.^19^^,^^20^ In this study, we excluded persons who reported a history of CVD or cancer at baseline (2,488 men and 3,187 women). We further excluded those with incomplete answers for the foods mainly contributing to dietary intakes in the food frequency questionnaire (FFQ) (20,742 men and 25,386 women) and those answers with implausibly high or low total energy intakes (370 men with energy intake <800 or >4,000 kcal/d and 78 women with energy intake <500 or >3,500 kcal/d).^21^ Ultimately, we included 22,795 eligible men and 35,539 eligible women from 45 communities in this study. The JACC Study was approved by the Ethics Boards at the Osaka University School of Medicine and the Nagoya University School of Medicine. Informed consents were obtained before participants completed the questionnaire or from community leaders instead of individuals.^20^

### Mortality

In each community, investigators conducted a systematic review of death certificates through the end of 2009, except in four areas where the follow-up had ended in 1999, another four areas where follow-up ended in 2003, and two areas where follow-up ended in 2008. In Japan, registration of death is legally required and is considered to be followed across Japan. In this cohort, all deaths occurred were ascertained by death certificates from a public health center, except for those who died after they had moved from their original communities, which were treated as censored when they moved out. The date of moving from the community was verified by population-register sheets. In total, there were 1,166 (5.1%) men and 2,191 (6.2%) women moved out of study areas during the follow-up period.

### Baseline questionnaire

The FFQ included 33 food items, with five choices for frequency of intake offered for each item.^22^ The amount of vitamin C, vitamin E, and main carotenoids that each food item contained was estimated based on the enlarged fifth version of the Japan Food Table.^23^ The amount of antioxidant nutrients were then calculated by multiplying the frequency scores by the estimated intakes of vitamin C (ascorbic acids), vitamin E (α-tocopherol), and carotenes from each food and summing across all 33 items, as has been validated previously.^22^ Intakes of tested antioxidant nutrients were adjusted for energy intake using the nutrient residual model.^24^ The mean energy-adjusted intake of vitamin C was underestimated by 30%, according to the validation study that compared them with dietary records in a subsample (*n* = 85, mostly female).^22^ Spearman’s correlation coefficient between vitamin C derived from the FFQ and dietary records was 0.38 for crude intake and 0.27 for energy-adjusted intake.^22^

### Statistical analysis

Person-years of follow-up were counted from the date of the survey until the time of death, the date of moving out of a study area, or the end of 2009, whichever came first. The mortality rates were calculated according to quintile intakes of energy-adjusted nutrients based on men and women cohorts, separately. Spearman correlations were performed to assess the collinearity among covariates, and participants’ characteristics were shown according to quintiles of vitamin C intake. Hazard ratios (HRs) with 95% confidence intervals (CIs) were computed with adjustment for age and other potential risk factors with Cox proportional hazards models, using the lowest quintile intake group as reference. The confounding factors included baseline body mass index (BMI; <20, 20 to <25, 25 to <30, and ≥30 kg/m^2^), smoking (never, former, current <20 cigarettes/d, and current ≥20 cigarettes/d), alcohol drinking (never, former, and current), daily walking time (never, 30 min to <1 h, and ≥1 h), weekly physical activity (never, 1–2, and ≥3 h), education years (<6, 6–10, 11–13, and >13 y), daily vitamin supplement using (no or yes), and menopausal status (no or yes [for women only]). Interaction was tested by including the factors of interest and the cross-product term in multivariate models.^25^ Associations of joint intakes of studied antioxidants with risk of all-cause mortality were measured using cross-products of tertile intakes of two antioxidants, which were categorized as low (both in tertile 1), intermediate (both in varying tertiles), and high (both in tertile 3).^26^ We ascertained dose-response trends by assigning median values to each exposure category and modeling these variables as continuous variables.^27^ Stratified analysis was performed for smoking status (current, former, and never) in participants. We used SAS version 9.4 (SAS Institute Inc, Cary, NC, USA) for the analyses. All probability values for statistical tests were two-tailed, and *P* values less than 0.05 were considered statistically significant.

## RESULTS

For tracking intakes of antioxidants, baseline characteristics of covariates in the study population were presented according to the energy-adjusted quintile intakes of vitamin C in Table 1. Smoking, alcohol consumption, weekly physical activities, and education level were higher in men than in women, while dietary intake of vitamin C, daily vitamin supplement use, and intakes of vegetable and fruit were higher in women than in men. Both in men and women, the vitamin C amount increased as intakes of vegetables and fruits increased. As the intake of vitamin C increased, the population of current smokers in men was decreased while the population of former smokers in men was slightly increased. Similar situations for current male smokers were observed according to other vitamins (data not shown). During a median follow-up of 18.9 years for 22,795 men and 19.4 years for 35,539 women, 6,179 and 5,355 deaths in men and women, respectively, were documented.

**Table 1.** Baseline characteristics of study population according to the energy-adjusted intake of vitamin C, JACC Study, 1988–1990

Characteristics	Quintiles of energy-adjusted intake of vitamin C^a^				

	Q1 (lower)	Q2	Q3	Q4	Q5
Men, *n*	4,559	4,559	4,559	4,559	4,559
Energy-adjusted vitamin C, median, mg/d	53	76	94	113	143
Age, years	54 (9.7)	55 (9.7)	55.8 (9.7)	56.8 (9.9)	58.1 (10.0)
BMI, kg/m^2^	22.7 (2.8)	22.7 (2.8)	22.7 (2.7)	22.7 (2.7)	22.7 (2.8)
Former smoker, %	21.9	23.0	24.6	25.8	26.2
Current smoker, %	58.9	54.6	52.7	50.1	47.4
Current drinker, %	77.9	76.9	76.5	75.1	71.7
Weekly physical activity ≥3 h, %	4.9	5.9	7.0	8.0	8.5
Daily walk ≥1 h, %	43.9	46.0	47.9	48.3	49.1
College education and above, %	14.1	15.8	16.7	18.6	19.3
Daily use of vitamin supplement, %	7.7	7.4	7.7	8.8	9.4
Daily dietary intake					
Total energy, kcal	1,771 (485)	1,805 (483)	1,812 (483)	1,812 (500)	1,708 (474)
Vegetable, g	47 (27)	73 (35)	91 (41)	112 (48)	145 (55)
Fruit, g	43 (36)	80 (49)	113 (61)	154 (71)	209 (80)

Women, *n*	7,107	7,108	7,108	7,108	7,108
Energy-adjusted vitamin C, median, mg/d	65	89	106	123	149
Age, years	55.4 (10.1)	55.4 (9.9)	56.2 (9.8)	56.6 (9.7)	57.8 (9.6)
BMI, kg/m^2^	23.0 (3.2)	22.9 (3.1)	22.9 (3.1)	22.9 (3.0)	23.0 (3.0)
Former smoker, %	1.3	1.3	1.2	1.2	1.4
Current smoker, %	6.3	4.6	3.8	3.8	4.0
Current drinker, %	23.2	22.6	22.6	22.0	21.3
Weekly physical activity ≥3 h, %	3.5	3.9	4.1	4.7	5.0
Daily walk ≥1 h, %	45.7	46.0	48.2	49.9	49.9
College education and above, %	7.1	9.0	10.2	11.0	11.6
Daily use of vitamin supplement, %	8.4	9.2	10.8	10.5	10.8
Daily dietary intake					
Total energy, kcal	1,416 (393)	1,447 (365)	1,442 (351)	1,450 (346)	1,391 (360)
Vegetable, g	62 (32)	89 (38)	105 (43)	125 (49)	153 (54)
Fruit, g	63 (43)	111 (55)	146 (62)	183 (70)	233 (76)

BMI, body mass index.

^a^Numbers are mean (SD) unless specified otherwise.

Table 2 shows the ranges and medians of dietary intake of antioxidant micronutrients, the number of deaths, followed person-years, and the associations between individual antioxidant nutrient intake and all-cause mortality in men and women, respectively. Comparing with women, men had lower intakes of vitamin C, α-carotene, β-carotene, and β-cryptoxanthin for each quintile. The correlation coefficients among vitamin C, vitamin E, α-carotene, and β-carotene (not shown in tables) ranged from 0.57–0.85 in men and from 0.52–0.84 in women, and the correlation coefficient between vitamin C and β-cryptoxanthin was 0.73 in men and 0.72 in women; lower coefficients were seen between β-cryptoxanthin and vitamin E (0.33 in men and 0.27 in women), α-carotene (0.17 in men and 0.11 in women), and β-carotene (0.24 in men and 0.18 in women).

**Table 2.** Ranges of energy-adjusted dietary antioxidant nutrients at baseline and hazard ratios for all-cause mortality during follow-up time in men and women, JACC Study

	Quintiles intakes of dietary antioxidant nutrients					*P*, trend

	Q1 (lower)	Q2	Q3	Q4	Q5	
Men						
Vitamin C						
Range, mg/d	<67	67–85	85–104	104–126	>126	
Median, mg/d	54	76	94	114	143	
Person-years	72,892	74,580	74,285	73,517	72,795	
Number of deaths	1,093	1,143	1,188	1,277	1,478	
Age-adjusted HR (95% CI)	1	0.89 (0.82, 0.97)	0.87 (0.80, 0.94)	0.86 (0.79, 0.93)	0.89 (0.83, 0.97)	0.016
Multivariate HR (95% CI)^a^	1	0.93 (0.86, 1.01)	0.93 (0.86, 1.01)	0.94 (0.87, 1.02)	0.97 (0.90, 1.05)	0.011
Vitamin E						
Range, mg/d	<4.1	4.1–4.7	4.7–5.3	5.3–6	>6	
Median, mg/d	3.7	4.4	5	5.6	6.5	
Person-years	73,343	74,884	74,602	73,918	71,320	
Number of deaths	1,039	1,079	1,154	1,324	1,583	
Age-adjusted HR (95% CI)	1	0.90 (0.83, 0.98)	0.89 (0.82, 0.97)	0.92 (0.85, 1.00)	0.97 (0.90, 1.05)	0.907
Multivariate HR (95% CI)^a^	1	0.98 (0.90, 1.07)	0.99 (0.91, 1.08)	1.03 (0.94, 1.12)	1.03 (0.95, 1.13)	0.808
α-carotene						
Range, µg/d	<164	164–226	226–295	295–397	>397	
Median, µg/d	122	196	258	338	494	
Person-years	72,361	74,079	74,374	74,059	73,195	
Number of deaths	985	1,089	1,183	1,381	1,541	
Age-adjusted HR (95% CI)	1	0.94 (0.87, 1.03)	0.94 (0.87, 1.03)	0.98 (0.91, 1.07)	1.00 (0.92, 1.08)	0.378
Multivariate HR (95% CI)^a^	1	0.98 (0.90, 1.07)	0.99 (0.91, 1.08)	1.03 (0.94, 1.12)	1.03 (0.95, 1.13)	0.789
β-carotene						
Range, µg/d	<1,301	1,301–1,782	1,782–2,294	2,294–2,959	>2,959	
Median, µg/d	1,139	1,739	2,291	2,918	3,892	
Person-years	73,480	74,899	74,154	74,103	71,432	
Number of deaths	982	1,112	1,224	1,287	1,574	
Age-adjusted HR (95% CI)	1	0.98 (0.90, 1.06)	0.97 (0.89, 1.05)	0.92 (0.84, 1.00)	0.98 (0.91, 1.07)	0.574
Multivariate HR (95% CI)^a^	1	1.02 (0.94, 1.11)	1.02 (0.93, 1.11)	0.97 (0.89, 1.06)	1.04 (0.95, 1.13)	0.309
β-cryptoxanthin						
Range, µg/d	<215	215–408	408–658	658–966	>966	
Median, µg/d	101	307	519	807	1,206	
Person-years	73,355	74,703	73,840	74,049	72,121	
Number of deaths	1,292	1,184	1,184	1,217	1,302	
Age-adjusted HR (95% CI)	1	0.87 (0.80, 0.94)	0.84 (0.77, 0.91)	0.83 (0.76, 0.89)	0.89 (0.83, 0.97)	0.017
Multivariate HR (95% CI)^a^	1	0.92 (0.85, 0.99)	0.89 (0.82, 0.96)	0.88 (0.81, 0.95)	0.97 (0.90, 1.05)	0.028
Women						
Vitamin C						
Range, mg/d	<78	78–97	97–114	114–134	>134	
Median, mg/d	65	88	106	123	149	
Person-years	116,969	118,072	118,656	119,845	120,213	
Number of deaths	1,094	967	1,063	1,059	1,172	
Age-adjusted HR (95% CI)	1	0.87 (0.80, 0.95)	0.86 (0.79, 0.94)	0.81 (0.75, 0.88)	0.80 (0.74, 0.87)	<0.0001
Multivariate HR (95% CI)^a^	1	0.89 (0.82, 0.97)	0.88 (0.81, 0.96)	0.84 (0.77, 0.92)	0.83 (0.76, 0.90)	<0.0001
Vitamin E						
Range, mg/d	<4.2	4.2–4.8	4.8–5.3	5.3–5.9	>5.9	
Median, mg/d	3.8	4.6	5.1	5.6	6.4	
Person-years	116,019	118,768	119,651	119,465	119,852	
Number of deaths	1,075	976	1,043	1,070	1,191	
Age-adjusted HR (95% CI)	1	0.90 (0.83, 0.99)	0.88 (0.80, 0.95)	0.84 (0.78, 0.92)	0.83 (0.76, 0.90)	<0.0001
Multivariate HR (95% CI)^a^	1	0.92 (0.84, 1.01)	0.91 (0.84, 1.00)	0.88 (0.81, 0.96)	0.85 (0.78, 0.93)	<0.0001
α-carotene						
Range, µg/d	<191	191–262	262–338	338–456	>456	
Median, µg/d	150	226	300	384	541	
Person-years	113,093	118,785	119,628	120,736	121,512	
Number of deaths	924	984	1,013	1,154	1,280	
Age-adjusted HR (95% CI)	1	0.95 (0.86, 1.03)	0.91 (0.83, 1.00)	0.92 (0.84, 1.00)	0.92 (0.84, 1.00)	0.084
Multivariate HR (95% CI)^a^	1	0.95 (0.87, 1.04)	0.93 (0.85, 1.02)	0.93 (0.85, 1.02)	0.93 (0.85, 1.02)	0.033
β-carotene						
Range, µg/d	<1,709	1,709–2,287	2,287–2,855	2,855–3,569	>3,569	
Median, µg/d	1,363	1,999	2,565	3,180	4,096	
Person-years	116,017	118,952	119,937	120,061	118,788	
Number of deaths	992	925	1,067	1,058	1,313	
Age-adjusted HR (95% CI)	1	0.85 (0.78, 0.93)	0.88 (0.81, 0.96)	0.78 (0.71, 0.85)	0.86 (0.79, 0.94)	0.0008
Multivariate HR (95% CI)^a^	1	0.86 (0.78, 0.94)	0.90 (0.83, 0.99)	0.82 (0.75, 0.89)	0.88 (0.81, 0.96)	0.0006
β-cryptoxanthin						
Range, µg/d	<327	327–581	581–841	840–1,101	>1,101	
Median, µg/d	204	450	731	953	1,342	
Person-years	120,305	119,626	120,141	118,247	115,436	
Number of deaths	1,174	1,104	1,054	1,040	983	
Age-adjusted HR (95% CI)	1	0.95 (0.88, 1.04)	0.87 (0.80, 0.94)	0.86 (0.79, 0.94)	0.86 (0.79, 0.94)	<0.0001
Multivariate HR (95% CI)^a^	1	0.99 (0.91, 1.07)	0.90 (0.83, 0.98)	0.90 (0.83, 0.98)	0.90 (0.82, 0.98)	0.0002

BMI, body mass index; CI, confidence interval; HR, hazard ratio.

^a^Adjusted for age, BMI, smoking habit, alcohol consumption, vitamin supplement use, daily walk, weekly physical activity, education level, study area, sleep disorder, and menopause status (for women).

In the age-adjusted model, higher intakes of vitamin C and β-cryptoxanthin were inversely associated with mortality risk in men, with statistically significant trends. In multivariate analysis, these associations were attenuated without reaching the significance level in the highest quintile (*P* for trend > 0.05). The association between β-cryptoxanthin intake and all-cause mortality showed a U-shape, with significant lower risk in the intermediate quintiles. In both age-adjusted and multivariate models, the dietary intakes of vitamin C, vitamin E, β-carotene, and β-cryptoxanthin among women were significantly associated with mortality risk, with 10–17% reductions in the fifth quintile compared with the first quintile of intakes (Table 2). When we added the dietary intake of fat for adjustment, the associations between studied nutrients and all-cause mortality risk were not altered (data not shown).

There were no statistically significant interactions effects observed between smoking and alcohol drinking, or between smoking or alcohol drinking and any micronutrients, by adding the cross-product in the univariate and multivariate models. Similarly, there were no significant interaction effects on all-cause mortality (*P* > 0.05) observed for any two of individual nutrients when simultaneously including them with their interaction term in the models. Therefore, Table 3 shows results of main effects from combined categories of two individual nutrients without the interaction term. For the nutrient-mortality associations based on their joint intakes, we observed 3–6% lower risk in the highest vs lowest categories of combined intakes of any two of vitamin C and carotenes in men and 12–17% lower risk in the highest vs lowest categories of combined intakes of any two of vitamin C, E, α-carotene, β-carotene, and β-cryptoxanthin (with distinctively significant trends) in women (Table 3).

**Table 3.** Joint effects of highly correlated antioxidant nutrients on all-causes mortality in men and women, JACC Study

	Dietary intakes of nutrients, men^a^				Dietary intakes of nutrients, women^a^			
	
	Low	Others	High	*P*, trend	Low	Others	High	*P*, trend
Vitamin C + Vitamin E								
Person-years	84,991	200,710	82,367		128,969	335,707	129,079	
Number of cases	1,214	3,239	1,726		1,192	2,898	1,265	
Age-adjusted HR (95% CI)	1	0.92 (0.86, 0.99)	0.95 (0.88, 1.02)	0.220	1	0.87 (0.81, 0.93)	0.80 (0.74, 0.87)	<0.0001
Multivariate HR (95% CI)^b^	1	0.97 (0.90, 1.03)	1.00 (0.93, 1.08)	0.134	1	0.90 (0.84, 0.96)	0.83 (0.76, 0.90)	<0.0001
Vitamin C + α-carotene								
Person-years	72,510	222,758	72,799		107,754	372,811	113,190	
Number of cases	1,011	3,636	1,532		938	3,235	1,182	
Age-adjusted HR (95% CI)	1	0.93 (0.87, 1.00)	0.93 (0.86, 1.01)	0.124	1	0.88 (0.82, 0.94)	0.83 (0.76, 0.90)	<0.0001
Multivariate HR (95% CI)^b^	1	0.96 (0.89, 1.03)	0.97 (0.89, 1.06)	0.049	1	0.89 (0.83, 0.96)	0.85 (0.78, 0.93)	<0.0001
Vitamin C + β-carotene								
Person-years	80,117	208,166	79,784		123,250	345,035	125,470	
Number of cases	1,137	3,377	1,665		1,075	2,984	1,296	
Age-adjusted HR (95% CI)	1	0.91 (0.85, 0.97)	0.91 (0.84, 0.98)	0.031	1	0.88 (0.82, 0.94)	0.83 (0.76, 0.90)	<0.0001
Multivariate HR (95% CI)^b^	1	0.94 (0.88, 1.01)	0.96 (0.89, 1.04)	0.013	1	0.91 (0.85, 0.98)	0.87 (0.80, 0.94)	<0.0001
Vitamin C + β-cryptoxanthin								
Person-years	81,601	201,965	84,502		135,238	328,180	130,337	
Number of cases	1,309	3,297	1,573		1,257	2,935	1,163	
Age-adjusted HR (95% CI)	1	0.88 (0.83, 0.94)	0.88 (0.81, 0.94)	0.001	1	0.86 (0.80, 0.92)	0.83 (0.77, 0.90)	<0.0001
Multivariate HR (95% CI)^b^	1	0.92 (0.86, 0.98)	0.94 (0.87, 1.02)	0.001	1	0.89 (0.83, 0.95)	0.87 (0.80, 0.95)	<0.0001
Vitamin E + α-carotene								
Person-years	83,849	202,534	81,684		126,639	335,453	131,663	
Number of cases	1,132	3,285	1,762		1,102	2898	1,355	
Age-adjusted HR (95% CI)	1	0.98 (0.92, 1.05)	1.02 (0.94, 1.10)	0.550	1	0.89 (0.83, 0.96)	0.86 (0.79, 0.93)	0.0002
Multivariate HR (95% CI)^b^	1	1.02 (0.95, 1.09)	1.06 (0.98, 1.15)	0.943	1	0.92 (0.86, 0.99)	0.88 (0.81, 0.96)	<0.0001
Vitamin E + β-carotene								
Person-years	93,870	182,599	91,599		145,702	299,426	148,627	
Number of cases	1,300	2,923	1,956		1,277	2,566	1,512	
Age-adjusted HR (95% CI)	1	0.94 (0.88, 1.00)	0.97 (0.91, 1.05)	0.647	1	0.88 (0.82, 0.94)	0.83 (0.77, 0.90)	<0.0001
Multivariate HR (95% CI)^b^	1	0.97 (0.91, 1.04)	1.03 (0.95, 1.10)	0.396	1	0.92 (0.86, 0.98)	0.87 (0.80, 0.94)	<0.0001
Vitamin E + β-cryptoxanthin								
Person-years	59,971	250,192	57,905		92,739	415,926	85,090	
Number of cases	896	4,093	1,190		869	3,704	782	
Age-adjusted HR (95% CI)	1	0.93 (0.87, 1.00)	0.94 (0.87, 1.03)	0.271	1	0.88 (0.82, 0.95)	0.82 (0.74, 0.90)	<0.0001
Multivariate HR (95% CI)^b^	1	0.97 (0.9, 1.04)	1.01 (0.93, 1.11)	0.223	1	0.93 (0.86, 1.00)	0.87 (0.79, 0.96)	<0.0001

CI, confidence interval; HR, hazard ratio.

^a^Low, all nutrients in tertile 1; high, all nutrients in tertile 3; intermediate, all nutrients in varying tertiles.

^b^Adjusted for age, body mass index, smoking habit, alcohol consumption, vitamin supplement use, daily walk, weekly physical activity, education level, study area, sleep disorder, and menopause status (for women).

For intensive control of smoking effect, we also conducted stratified analysis by smoking status, and there were no significant associations observed in male current, former, or never smokers (Table 4). However, statistically significant HRs were seen in the highest compared with the lowest quintiles of vitamin C, vitamin E, and β-carotene in female nonsmokers, but not in female former and current smokers (Table 5). Similar estimates were obtained when results were stratified by alcohol drinking (current, former, and never) or BMI (<25 and ≥25 kg/m^2^): the only significantly reduced risks were observed in female nondrinker or women with BMI <25 kg/m^2^. In female nondrinkers, reduced mortality risk was seen in the 5th vs 1st quintiles of vitamin C (HR 0.80; 95% CI, 0.72–0.88; *P* for trend < 0.001), vitamin E (HR 0.84; 95% CI, 0.76–0.92; *P* for trend < 0.001), α-carotene (HR 0.89; 95% CI, 0.80–0.99; *P* for trend = 0.103), β-carotene (HR 0.87; 95% CI, 0.79–0.96; *P* for trend = 0.021) and β-cryptoxanthin (HR 0.90; 95% CI, 0.81–0.99; *P* for trend = 0.004). In women with BMI <25 kg/m^2^, reduced mortality risk was seen in the 5th vs 1st quintiles of vitamin C (HR 0.79; 95% CI, 0.72–0.89; *P* for trend < 0.001), vitamin E (HR 0.83; 95% CI, 0.75–0.92; *P* for trend < 0.001), β-carotene (HR 0.87; 95% CI, 0.78–0.97; *P* for trend = 0.015) and β-cryptoxanthin (HR 0.89; 95% CI, 0.80–0.99; *P* for trend = 0.005).

**Table 4.** Associations between energy-adjusted dietary antioxidant nutrients and all-cause mortality in men of current, former and never smokers, JACC Study

	HR (95% CI) by quintile intakes of nutrients^a^				*P*, trend

	Q2	Q3	Q4	Q5	
Current smokers					
Vitamin C					
Age-adjusted	0.94 (0.85, 1.04)	0.92 (0.83, 1.02)	0.91 (0.82, 1.00)	0.97 (0.87, 1.07)	0.498
Multivariate^b^	0.95 (0.86, 1.05)	0.94 (0.85, 1.04)	0.93 (0.84, 1.03)	0.98 (0.89, 1.09)	0.730
Vitamin E					
Age-adjusted	0.92 (0.83, 1.02)	0.96 (0.86, 1.06)	0.91 (0.83, 1.01)	1.01 (0.92, 1.12)	0.686
Multivariate^b^	0.93 (0.84, 1.03)	0.97 (0.87, 1.07)	0.92 (0.83, 1.02)	1.01 (0.91, 1.12)	0.763
α-carotene					
Age-adjusted	0.97 (0.87, 1.08)	0.95 (0.86, 1.05)	0.98 (0.89, 1.09)	1.01 (0.91, 1.12)	0.573
Multivariate^b^	0.97 (0.87, 1.08)	0.96 (0.86, 1.06)	0.97 (0.87, 1.07)	0.99 (0.90, 1.10)	0.912
β-carotene					
Age-adjusted	1.02 (0.92, 1.13)	0.95 (0.85, 1.05)	0.98 (0.88, 1.08)	1.03 (0.93, 1.14)	0.720
Multivariate^b^	1.02 (0.92, 1.13)	0.96 (0.86, 1.06)	0.99 (0.89, 1.10)	1.01 (0.91, 1.12)	0.960
β-cryptoxanthin					
Age-adjusted	0.91 (0.83, 1.00)	0.84 (0.76, 0.93)	0.85 (0.77, 0.93)	0.98 (0.89, 1.08)	0.601
Multivariate^b^	0.92 (0.84, 1.02)	0.86 (0.78, 0.95)	0.86 (0.78, 0.95)	1.01 (0.92, 1.12)	0.992

Former smokers					
Vitamin C					
Age-adjusted	0.85 (0.72, 1.00)	0.85 (0.73, 1.00)	0.87 (0.75, 1.01)	0.92 (0.79, 1.06)	0.707
Multivariate^b^	0.87 (0.74, 1.02)	0.88 (0.75, 1.03)	0.90 (0.77, 1.05)	0.94 (0.81, 1.09)	0.937
Vitamin E					
Age-adjusted	0.93 (0.78, 1.10)	0.87 (0.74, 1.03)	0.97 (0.83, 1.13)	1.02 (0.88, 1.19)	0.284
Multivariate^b^	0.94 (0.79, 1.11)	0.87 (0.74, 1.02)	0.97 (0.82, 1.14)	1.01 (0.86, 1.18)	0.438
α-carotene					
Age-adjusted	1.02 (0.86, 1.21)	1.10 (0.93, 1.30)	0.99 (0.84, 1.16)	1.20 (1.03, 1.41)	0.012
Multivariate^b^	1.03 (0.87, 1.22)	1.11 (0.94, 1.31)	0.98 (0.83, 1.15)	1.15 (0.98, 1.34)	0.104
β-carotene					
Age-adjusted	1.01 (0.85, 1.20)	1.02 (0.86, 1.20)	0.94 (0.80, 1.11)	1.03 (0.88, 1.21)	0.823
Multivariate^b^	1.00 (0.84, 1.19)	1.03 (0.87, 1.21)	0.94 (0.79, 1.10)	1.00 (0.85, 1.17)	0.799
β-cryptoxanthin					
Age-adjusted	0.87 (0.74, 1.01)	0.82 (0.71, 0.96)	0.87 (0.75, 1.01)	0.85 (0.74, 0.99)	0.139
Multivariate^b^	0.88 (0.76, 1.03)	0.81 (0.70, 0.95)	0.89 (0.77, 1.04)	0.88 (0.76, 1.02)	0.325

Never smokers					
Vitamin C					
Age-adjusted	0.97 (0.78, 1.20)	0.91 (0.74, 1.13)	1.00 (0.81, 1.22)	0.95 (0.78, 1.16)	0.787
Multivariate^b^	0.98 (0.79, 1.22)	0.93 (0.75, 1.15)	1.02 (0.83, 1.25)	0.96 (0.78, 1.17)	0.810
Vitamin E					
Age-adjusted	0.92 (0.74, 1.15)	1.02 (0.83, 1.27)	0.95 (0.77, 1.18)	1.00 (0.82, 1.22)	0.764
Multivariate^b^	0.93 (0.75, 1.16)	1.03 (0.83, 1.27)	0.98 (0.79, 1.21)	1.01 (0.83, 1.23)	0.708
α-carotene					
Age-adjusted	0.92 (0.73, 1.16)	0.96 (0.77, 1.20)	1.14 (0.92, 1.42)	1.09 (0.88, 1.34)	0.073
Multivariate^b^	0.92 (0.73, 1.16)	0.96 (0.77, 1.21)	1.12 (0.90, 1.39)	1.04 (0.84, 1.29)	0.235
β-carotene					
Age-adjusted	0.90 (0.72, 1.13)	1.04 (0.84, 1.29)	0.87 (0.71, 1.08)	1.01 (0.82, 1.23)	0.778
Multivariate^b^	0.95 (0.76, 1.19)	1.07 (0.86, 1.32)	0.89 (0.72, 1.10)	1.02 (0.83, 1.25)	0.927
β-cryptoxanthin					
Age-adjusted	0.85 (0.70, 1.04)	0.91 (0.75, 1.10)	0.88 (0.73, 1.07)	0.95 (0.78, 1.15)	0.996
Multivariate^b^	0.85 (0.70, 1.04)	0.91 (0.75, 1.11)	0.91 (0.75, 1.10)	0.96 (0.79, 1.16)	0.828

CI, confidence interval; HR, hazard ratio.

^a^The lowest quintile (Q1) was used as the reference category (HR = 1).

^b^Adjusted for age, body mass index, alcohol consumption, vitamin supplement use, daily walk, weekly physical activity, education level, study area, and sleep disorder.

**Table 5.** Associations between energy-adjusted dietary antioxidant nutrients and all-cause mortality in women of current, former and never smokers, JACC Study

	HR (95% CI) by quintile intakes of nutrients^a^				*P*, trend

	Q2	Q3	Q4	Q5	
Current smokers					
Vitamin C					
Age-adjusted	0.91 (0.67, 1.22)	0.79 (0.57, 1.10)	0.63 (0.45, 0.88)	0.87 (0.64, 1.16)	0.092
Multivariate^b^	0.90 (0.67, 1.22)	0.83 (0.59, 1.15)	0.64 (0.45, 0.91)	0.88 (0.65, 1.19)	0.130
Vitamin E					
Age-adjusted	0.94 (0.70, 1.26)	0.93 (0.67, 1.30)	0.92 (0.67, 1.26)	0.92 (0.68, 1.26)	0.563
Multivariate^b^	0.94 (0.70, 1.27)	0.93 (0.66, 1.30)	0.92 (0.67, 1.27)	0.90 (0.66, 1.23)	0.487
α-carotene					
Age-adjusted	0.76 (0.56, 1.05)	0.91 (0.66, 1.24)	0.95 (0.70, 1.29)	0.88 (0.64, 1.21)	0.784
Multivariate^b^	0.81 (0.59, 1.12)	0.91 (0.66, 1.25)	0.93 (0.68, 1.27)	0.87 (0.63, 1.19)	0.574
β-carotene					
Age-adjusted	0.84 (0.62, 1.15)	1.12 (0.82, 1.52)	0.70 (0.49, 1.00)	0.96 (0.71, 1.29)	0.631
Multivariate^b^	0.86 (0.63, 1.17)	1.11 (0.81, 1.52)	0.73 (0.51, 1.05)	0.95 (0.70, 1.29)	0.615
β-cryptoxanthin					
Age-adjusted	0.94 (0.69, 1.27)	0.79 (0.57, 1.10)	0.75 (0.54, 1.03)	0.81 (0.60, 1.09)	0.080
Multivariate^b^	0.89 (0.65, 1.22)	0.83 (0.59, 1.15)	0.79 (0.57, 1.10)	0.83 (0.61, 1.12)	0.169

Former smokers					
Vitamin C					
Age-adjusted	0.83 (0.47, 1.48)	0.87 (0.48, 1.56)	1.00 (0.58, 1.73)	0.72 (0.40, 1.29)	0.443
Multivariate^b^	0.90 (0.50, 1.63)	0.83 (0.45, 1.52)	0.96 (0.55, 1.70)	0.67 (0.36, 1.24)	0.282
Vitamin E					
Age-adjusted	1.23 (0.69, 2.21)	1.07 (0.60, 1.93)	1.05 (0.62, 1.78)	0.73 (0.43, 1.25)	0.111
Multivariate^b^	1.30 (0.71, 2.38)	1.11 (0.60, 2.07)	1.08 (0.62, 1.88)	0.65 (0.37, 1.16)	0.128
α-carotene					
Age-adjusted	1.00 (0.55, 1.82)	1.07 (0.57, 1.99)	1.29 (0.69, 2.41)	1.03 (0.56, 1.86)	0.036
Multivariate^b^	0.84 (0.45, 1.57)	0.73 (0.37, 1.42)	1.33 (0.69, 2.56)	0.71 (0.38, 1.34)	0.548
β-carotene					
Age-adjusted	1.25 (0.71, 2.19)	0.68 (0.35, 1.30)	1.02 (0.57, 1.82)	0.47 (0.23, 0.98)	0.030
Multivariate^b^	0.64 (0.33, 1.22)	1.01 (0.54, 1.86)	1.01 (0.55, 1.84)	0.63 (0.34, 1.18)	0.399
β-cryptoxanthin					
Age-adjusted	0.78 (0.42, 1.44)	1.13 (0.63, 2.01)	1.04 (0.58, 1.85)	0.83 (0.47, 1.49)	0.802
Multivariate^b^	0.88 (0.51, 1.52)	0.76 (0.41, 1.39)	1.04 (0.58, 1.87)	0.71 (0.39, 1.26)	0.333

Never smokers					
Vitamin C					
Age-adjusted	0.89 (0.81, 0.98)	0.89 (0.81, 0.97)	0.84 (0.77, 0.92)	0.84 (0.77, 0.91)	<0.0001
Multivariate^b^	0.91 (0.83, 0.99)	0.92 (0.84, 1.00)	0.89 (0.81, 0.97)	0.88 (0.80, 0.96)	0.005
Vitamin E					
Age-adjusted	0.86 (0.78, 0.94)	0.85 (0.78, 0.93)	0.84 (0.77, 0.92)	0.82 (0.75, 0.89)	<0.0001
Multivariate^b^	0.88 (0.80, 0.96)	0.88 (0.81, 0.96)	0.88 (0.80, 0.96)	0.85 (0.78, 0.92)	0.001
α-carotene					
Age-adjusted	0.99 (0.90, 1.09)	0.92 (0.84, 1.01)	0.93 (0.85, 1.02)	0.95 (0.87, 1.04)	0.285
Multivariate^b^	0.99 (0.90, 1.09)	0.93 (0.84, 1.02)	0.92 (0.84, 1.01)	0.96 (0.87, 1.05)	0.275
β-carotene					
Age-adjusted	0.87 (0.79, 0.96)	0.88 (0.80, 0.96)	0.80 (0.73, 0.87)	0.89 (0.81, 0.97)	0.012
Multivariate^b^	0.88 (0.80, 0.97)	0.89 (0.82, 0.98)	0.83 (0.76, 0.91)	0.91 (0.83, 0.99)	0.062
β-cryptoxanthin					
Age-adjusted	0.97 (0.89, 1.06)	0.87 (0.80, 0.95)	0.91 (0.83, 0.99)	0.89 (0.82, 0.98)	0.004
Multivariate^b^	0.98 (0.90, 1.07)	0.91 (0.83, 0.99)	0.95 (0.87, 1.04)	0.93 (0.85, 1.02)	0.098

CI, confidence interval; HR, hazard ratio.

^a^The lowest quintile (Q1) was used as the reference category (HR = 1).

^b^Adjusted for age, body mass index, alcohol consumption, vitamin supplement use, daily walk, weekly physical activity, education level, study area, sleep disorder, and menopause status.

When 1,862 (8.2%) men and 3,530 (9.9%) women who used daily supplements of multivitamin, vitamin C, or vitamin E were totally or respectively excluded from the analysis, the results of the associations between individual or combined antioxidant nutrients and all-cause mortality were similar to those in Table 2 and Table 3 (data not shown). Exclusion of deaths (476 in men and 326 in women) in the first 3 years of follow-up did not substantially change the findings. The similar insignificant associations with all-cause mortality were observed in men, except the U-shaped association for β-cryptoxanthin with significant association in the intermediate quintiles (HR 0.90; 95% CI, 0.83–0.98 in the 2nd quintile, HR 0.87; 95% CI, 0.80–0.95 in the 3rd quintile, and HR 0.88; 95% CI, 0.81–0.95 in the 4th quintile; *P* for trend = 0.032). Meanwhile, more strongly significant associations for vitamin C, vitamin E, β-carotene, and β-cryptoxanthin (*P* for trend < 0.001) were seen in women (data not shown). In further analysis stratified by smoking status, when removing daily vitamin supplements use, death within the first 3 years of follow-up, or both, these significant associations in women nonsmokers remained, and inverse associations in intermediate quintiles of β-cryptoxanthin in men was also observed (data not shown).

## DISCUSSION

In this large prospective cohort study, increased dietary intakes of vitamin C, vitamin E, β-carotene, and β-cryptoxanthin were associated with reduced all-cause mortality in middle-aged Japanese women. These significant inverse associations were prominently observed in female nonsmokers. These results provide evidence in Japanese population that is consistent with the findings from non-Asian population-based cohort studies on vitamin C,^6^^,^^7^ vitamin E,^10^ α-carotene,^7^ β-carotene,^7^^,^^10^ and β-cryptoxanthin.^7^ The inverse associations for individual micronutrients and the strong inverse associations for combined dietary intakes of antioxidants may represent joint effects of highly correlated micronutrients.^28^

Dietary intakes of vitamin C, vitamin E, and β-carotene were higher in women than those in men in this study. Men tend to underreport their dietary intake when it is ascertained through a FFQ,^29^ and women might recall dietary habits better than men, given their traditional roles in buying food and cooking.^2^ For dietary habit, individuals who consume more fruits and vegetables may also consume less dietary fat or may be more health-conscious in other ways than individuals who consume relatively few fruits and vegetables.^30^ This may be related to the better micronutrient density usually observed in women than in men and to risky lifestyles observed more in men than in women, such as smoking, drinking alcoholic beverages, or being overweight, which are known to decrease β-carotene and vitamin C serum levels.^31^ Although these variables were adjusted in the analyses, residual confounders or unmeasured variables could account for the discrepancy. The higher mortality from CVD and non-hormone-dependent cancers in Japanese men compared with women^14^^,^^19^ might be partially explain the disparities of associations observed in men and women in this study population. The JACC Study reported the inverse associations of dietary intakes of vitamins C and E with mortality from CVD,^14^ dietary intake of α-carotene with mortality from prostate cancer,^32^ and serum carotenes with mortality from all cancer,^18^ colorectal cancer,^18^ and lung cancer.^33^ However, the associations of antioxidant nutrient intakes with mortality from all cancer and many other types of cancer need to be further investigated in the JACC Study.^18^^,^^32^ Combining all cancer sites may dilute the observed associations regarding specific cancers, and the protective effect on all-cause mortality from dietary intakes of antioxidant nutrients may be mainly dependent on reductions in CVD mortality rather than cancer mortality.^9^

A Spanish study reported significant lower mortality in those with higher dietary β-cryptoxanthin intake,^7^ and, in a Dutch elderly population, significantly higher risk of all-cause mortality was observed in the lower serum β-cryptoxanthin group.^34^ Beta-cryptoxanthin, lutein, and zeaxanthin are oxygenated carotenoids.^34^ In our study, the U-shaped association between the dietary intake of β-cryptoxanthin and all-cause mortality in men and null results of an α-carotene-mortality relationship in women may indicate the complex mechanism of carotenoids in antioxidant activities. For instance, β-cryptoxanthin scavenges for free radicals in a polar environment, while α-carotene clears more deep in the lipoprotein cell membrane layer.^35^ It is also possible that carotenes are potentially acting as markers for other correlated etiologic factors.^30^^,^^36^ It is unclear when these dietary components are most effective in the prevention of disease, although we did not observe any significant interaction effects among studied micronutrients. Whether there is a threshold or dose-response effect, or even a triage effect from carotenoids, may depend on particular outcomes of diseases, including CVD, cancer, metabolic syndrome, or dysglycemia.^37^ Nevertheless, these different findings among studies might be due to the disparities of study design, populations, main causes of death, and also food sources or components.^38^^,^^39^

Smoking increases the utilization of antioxidant micronutrients on the basis of increased oxidative stress, which contributes to the low plasma antioxidant concentration.^40^ The increased oxidative stress enhances the possibility of gene mutations, oxidization of lipids and proteins, and alteration of signal transduction pathways that damage cells.^41^^,^^42^ Our study results were consistent with other reports that the dietary intakes of vitamin C, vitamin E, and α- and β-carotene were higher in nonsmokers than in smokers.^43^ A study in French women showed that, in never smokers, increasing dietary β-carotene intake was associated with a decreased risk of tobacco-related cancer.^44^ Smoking causes oxidative damage in organs, so smokers may benefit from intake of foods rich in antioxidant for reducing the risk of disease occurrences.^11^ However, in our study, those insignificant associations both in male and female smokers/former smokers also indicated that the protective effect of antioxidant micronutrients may not be strong enough to counteract the increased oxidative stress from tobacco smoking.^45^ Dietary modification should not be considered a substitute for smoking prevention.^25^

In this study population, the daily use of vitamin supplement (8.2% in men and 9.9% in women) was lower than in other populations (eg, 61–68% in the United States).^6^ Carotenoids, such as β-carotene, may have a possible biphasic response that promotes health when taken at dietary levels but may have adverse effects when taken in higher amounts.^38^ Dietary supplements of some carotenoids have been associated with increased risks of degenerative diseases^46^ and lung cancer.^47^ Studies also found consistent evidence of deleterious effects on all-cause mortality for dietary supplements containing vitamin A, vitamin E, and/or β-carotene.^10^^,^^48^ The similar significant inverse associations observed after removing the daily vitamin supplement in this study elucidated that the significant associations of nutrient intakes and all-cause mortality were driven by basic dietary intakes rather than supplements.

The strengths of this study include its prospective cohort design, a large number of deaths during a long follow-up time with sufficient statistical power, multiple adjustments for relevant confounders, and evaluation of an Asian population. The limitations of this study warrant discussion. First, we used an FFQ with only 33 food items to identify intakes of antioxidants, and we used death certificates to define events. The validity of FFQ for intakes of antioxidant vitamins with diet record was not high, and we did not have data about vitamin E and carotene in the validation study.^22^ However, the diet misclassification would attenuate findings toward the null in this study, since it is unlikely to be related to the baseline intakes of antioxidant vitamins. Second, the exclusion of missing dietary information may affect generalizability, although it may not greatly affect the significant findings in this study.^22^ Third, the possibility of other confounding by unmeasured or incompletely adjusted all-cause mortality risk factors also applies to this study, including the residual confounding by smoking.^25^^,^^36^ Fourth, because we only conducted a FFQ survey for baseline dietary intake, changes in vitamin intakes over time were not accessed to examine temporal aspects.^27^

Results of this study suggest that dietary intakes of vitamin C, vitamin E, β-carotene, and β-cryptoxanthin were inversely associated with all-cause mortality in Japanese women. These findings are consistent with the notion that intake of fruits and vegetables that are rich in dietary antioxidant micronutrients may be independently or jointly protective against all-cause mortality, particularly in female nonsmokers. Further studies on modified effects from lifestyle factors on the nutrient-mortality relationship are needed.
